# Bubble formation and scale dependence in free-surface air entrainment

**DOI:** 10.1038/s41598-019-46883-5

**Published:** 2019-07-29

**Authors:** Wangru Wei, Weilin Xu, Jun Deng, Zhong Tian, Faxing Zhang

**Affiliations:** State Key Lab of Hydraulic and Mountain River Engineering, Sichuan University, Chengdu, 610065 China

**Keywords:** Civil engineering, Fluid dynamics

## Abstract

The air bubble entrainment and self–aeration phenomena in free-surface water flows reveal a rich interplay of fundamental science and engineering, and the size distribution of the entrained bubbles enhances the air–water gas flux, improves the gas transfer, and influences the cavitation erosion protection in high–speed flows. In the present study, we investigate the bubble–formation mechanism of free–surface air entrainment and the related microscopic bubble scale in the laboratory. This paper provides a quantitative description of bubble entrainment. The entrapment deformation of the local free surface over a period follows a power–law scaling and entrains a bubble when the entrapped surface becomes enclosed in the unstable movement period. Both the size scale and shape character of the entrapped free surface determine the size and skewness of the distribution of the air bubble. The entrapment deformation process confirms that the instability behaviour of the local air–water interface results in the onset of bubble entrainment. Further research is necessary to elucidate the instability criterion dominated by the interface instability and promote a new understanding of multiphase flow generation and development.

## Introduction

Free–surface air entrainment in a high–speed water flow creates dense plumes of bubbles that can diffuse across a full cross-section; it has air quantity or void fractions exceeding 60–70%. Bubbles in the self–aerated region range in size from the sub–millimetre scale to tens of centimetres and are known to play important roles in a diverse set of phenomena, including an increase in the mixture flow bulk, a reduction in the cavitation erosion, and an enhancement in the gas transport across the flow^1–3^. Moreover, the variation in the viscosity of the air–water mixture volume in the stratification layer can alter the inflection point in the velocity and turbulence profile^4^. The most important issue of free–surface air entrainment is the mechanism of single bubble formation. Some studies have fairly analysed the basic dynamics of free-surface properties in open channels^5–7^. However, until now, few field measurements of air entrainment during the first tens of milliseconds of free–surface deformation have been reported, and bubble entrainment mechanisms have not been quantified. One of the problems with the existing measurements of microscopic free–surface air entrainment was that the local microscopic free surface evolved rapidly in the first milliseconds, complicating the reconciliation of different air-entrainment sets without knowing the exact age of the bubble formations.

When the free–surface turbulent intensity exceeded a critical fluctuation velocity, the theory of the “bubble being created by a water droplet” posited that bubble entrainment resulted from the water droplet falling back and impacting the free surface^8,9^ and that self-aerated flows contained a “water droplet layer” and an “air bubble layer”^10,11^. Based on this mechanism, a series of critical hydraulic conditions for the inception of self-aeration were deduced^12–14^, which were related to the flow depth, channel slope and wall roughness. However, Rein^15^ discussed water–droplet formation in self–aerated open channel flows, indicating that this theory did not explain the ejection of droplets to the heights that were observed in experiments. Combined with subsequent research on air–water structures^16,17^, which showed that “entrapped air near the distorted free surface and the entrained bubble were the two main air types,” Rein’s^15^ discussion led to the conclusion that droplet impact was not the only mechanism of air entrainment. Falvey and Ervine^18^ discussed the formation of entrained air bubbles under the assumption that the bubble creation depended on the enclosure between distorted free surfaces. In addition, significant investigations into the free surface in open channel flows^19,20^ indicated that the process of free–surface entrapment in water flow was the most important feature of air entrainment for all self-aeration phenomena. Wei *et al*.^21^ discussed the radius of curvature in the free–surface entrapped deformation and described a rapid evolution, which can be considered a critical shape condition for air entrainment. However, because of the complexity of air–water interactions, the temporal and spatial scales in the air–entrainment process were still insufficiently understood, and achieving coherent recognition of the free–surface air–entrainment mechanism was difficult. In the present study, we use optical high–speed observations of detailed free–surface deformation processes to quantify various aspects of the phenomena to determine the air entrainment.

## Results

### Origin of self-aeration

When the free–surface turbulent intensity in open-channel flows exceeds a critical fluctuation velocity to overcome gravity and the surface tension restraint, the lifetime of a surface–deformation–generated bubble falls into two periods. The first period occurs as a smooth free surface becomes rough and an air cavity forms between local entrapped surfaces. The size of the local entrapped deformation develops gradually, and the free–surface roughness increases. This period is accompanied by an interaction among multiple forces, including turbulent stresses, surface tension and gravity. The second period occurs when the forces on the local surface cannot balance, the deformation process ceases, and air entrainment is induced. Here, the physical process of free–surface entrapment deformation before the bubble formation is described, and the air–water interface transition between the free surface and air bubble is determined.

**Figure 1:**
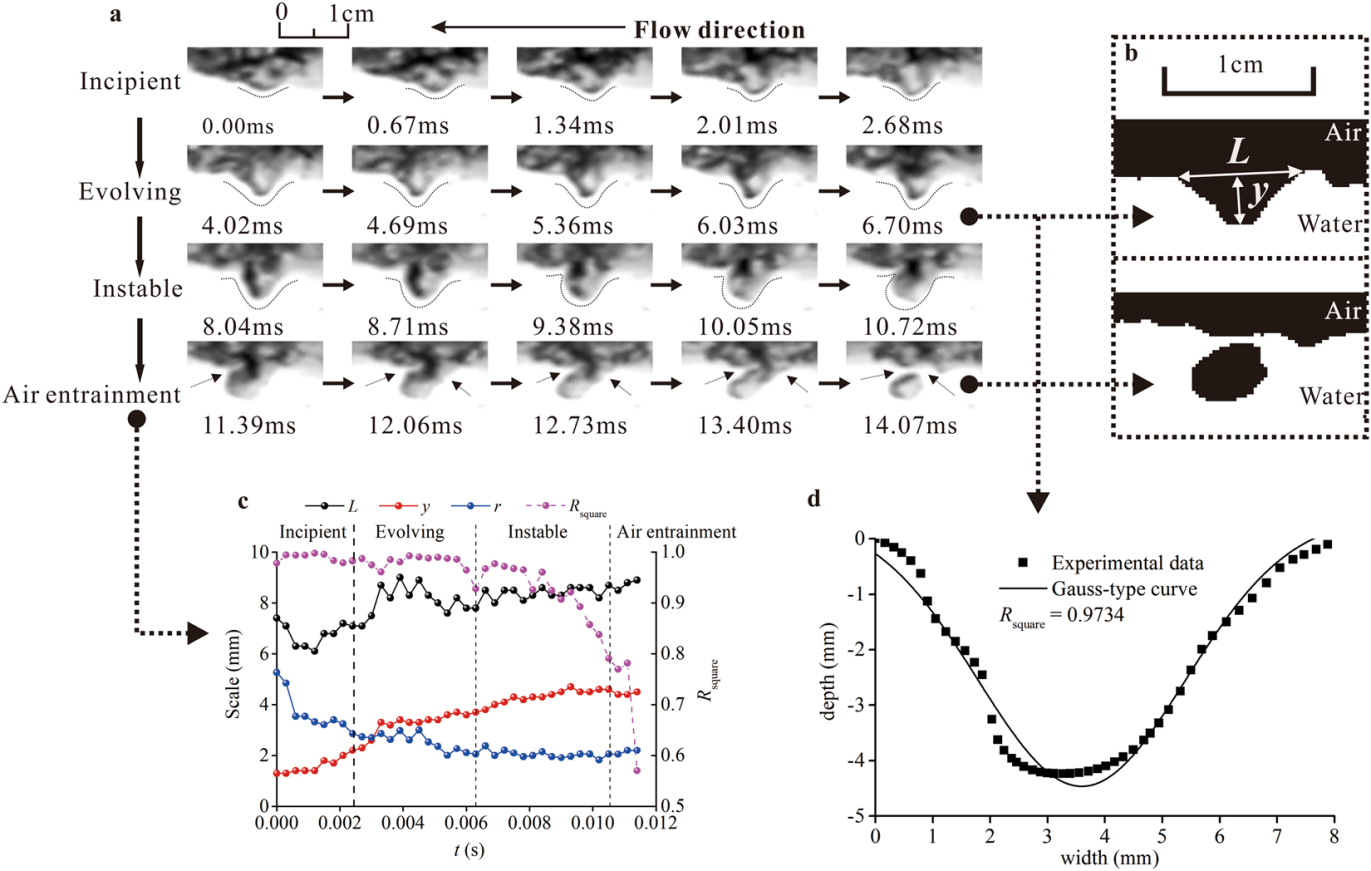
Free-surface entrapped deformation and bubble–entrainment process. (**a**) High–speed video images of the entire life of the free surface entrapment deformation and air entrainment process. Image sequences are captured at 1/3000 s with 1280 (width) × 344 (height) pixels and a spatial resolution of approximately 4 pixels/mm. The shutter speed is 0.23 μs. (**b**) Binary image for determining the length scale of the entrapment surface and entrained air bubble. (**c**) Size scales of the width *L*, depth *y*, and radius of curvature *r* of the entrapped surface throughout the entire lifetime, including the incipient, evolving, instable and air entrainment periods. The radius of curvature at the apex of the entrapped surface *r* is defined from mathematics^39^ as *r* = 0.125·*L*·(*L*/*y*). The right axis shows the corresponding correlation coefficient *R*_square_ of the Gaussian–type curve for the entrapment deformation. (**d**) Comparison of the entrapment deformation with the Gaussian–type curve.

The deformed free surface evolves over a range of lengths and time scales. The flow features forming distinct and repeatable patterns over the entire lifetime of the free surface air entrainment process are shown in Fig. 1. The entire process includes four periods:

- An incipient period, during which the local entrapped shape of the local surface slightly deforms but the cavity of the air entrapped between two sides of the free surface is still weakly developed.
- An evolving period, during which the entrapped cavity is almost completely developed, and the apex has reached the deepest distance in the water, with the two sides of the free surface remaining smooth and intact.
- An instable period, in which the whole entrapment of the free surface completely loosens, and the shape of the cavity collapses compared to its shape in the first two periods.
- An air–entrainment period, during which shrinkages appear on the two sides of the entrapped free surface as the unstable deformation develops further. These shrinkages develop longitudinally and ultimately penetrate the entrapped cavity. This process represents the enclosing of the free surfaces and the releasing of a single–formed air bubble in the water flow.

**Figure 2:**
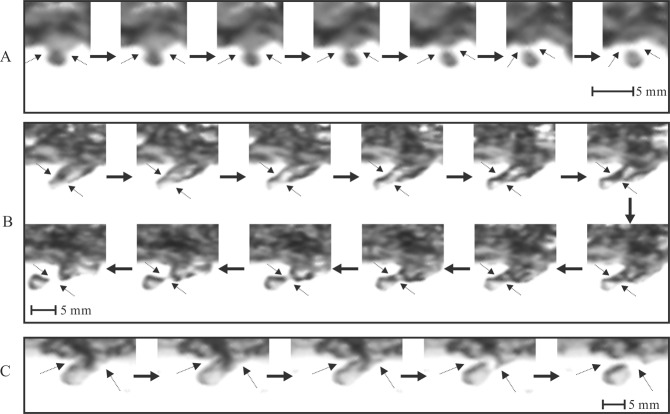
Three groups of air entrainment through the free–surface instability and enclosure with consecutive images (the time interval between two images is 0.33 ms): A, *d*_ab_ = 2.5 mm; B, *d*_ab_ = 4.3 mm; and C, *d*_ab_ = 8.6 mm.

The images in Fig. 1 suggest two distinct free-surface features driving air-bubble creation: entrapped deformation and cavity instability. This supports the analysis of air entrainment within the interaction of free surfaces^22,23^. In the air entrainment period, the intense and rapid change of the unstable surface occurs in an extremely short period (approximately 1–5 ms). The consecutive images recorded in the final period before the single bubble is generated are shown in Fig. 2. The observed enclosure positions and directions are obviously distinguished, which is dependent on the sharp movement of the entrapped surface during the instable period. On the other hand, a necking type of the entrapped cavity caused by dynamic shrinkage is the main surface behaviour. The shrinkage position remains approximately unchanged until the two-side surfaces finally close, releasing the entrained bubble into the water flow. Newly created air bubbles are considered as cavity remnants.

Assuming that the entrapped shape of the free surface is a Gaussian–type curve (Fig. 1(b,d)), the scale and shape variations throughout the lifetime of the air entrainment are displayed in Fig. 1(c). In the incipient and evolving periods, the correlation coefficient of the Gaussian curve *R*_square_ remains greater than 0.95, and the width *L* and depth *y* noticeably increase with increasing deformation intensity, which is represented by the decrease in the radius of curvature of the apex *r*. These results indicate that the entrapment intensity develops gradually and that the shape of the surface deformation remains stable. The instable period is characterized by a sharp decrease in *R*_square_ without remarkable changes in the other parameters of the entrapped deformation. Notably, in the late instable period, *R*_square_ < 0.80, indicating that the entrapped cavity almost collapses and the entrapped shape can no longer be considered as a Gaussian curve in the ensuing air entrainment process. The first image in which the *R*_square_ decreases below 0.80 is considered as the critical point of the transition between the “instable” and “air entrainment” periods, because the shape of the entrapment deformation finally collapses in the instable period and the single bubble is quickly generated. Based on the continuous images recorded by the high–speed camera, the beginning of the instable period is determined by the first image in which the *R*_square_ decreases below 0.95. The critical sizes of the width *L*_C_, depth *y*_C_ and radius of curvature *r*_C_ are obtained from average values of the first five images in the instable period.

**Figure 3:**
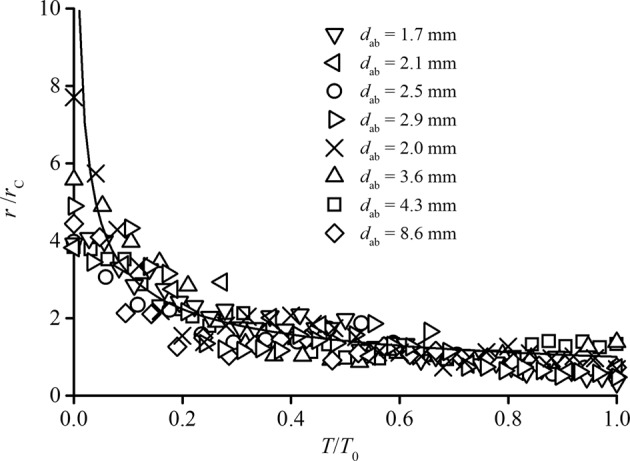
Variation in the radius of curvature in the free-surface deformation process. The ratio of *r* to critical value *r*_C_ in the air entrainment lifetime was obtained from eight bubble entrainment events. The equivalent diameter of the bubble size *d*_ab_ ranged from 1.7 to 8.6 mm; each bubble was considered to be a spherical particle with identical areas.

Figure 3 shows the results of image analysis over eight air–entrainment events, plotted as the radius of curvature *r*/*r*_C_ versus time *T*/*T*_0_, where *T* is the local time and *T*_0_ is the total time among the incipient, evolving, and instable periods. The *r*/*r*_C_ spectrum shows an approximately identical scaling law:

1 $$\frac{r}{{r}_{{\rm{C}}}}={(\frac{T}{{T}_{0}})}^{-0.5}$$

The upper asymptotic value of *r*/*r*_C_ → ∞ for *T*/*T*_0_ → 0 represents the initial state of the free surface entrapped deformation. In addition, *r*/*r*_C_ → 1 for *T*/*T*_0_ → 1, implying that critical *r*_C_ appears for certain water–flow conditions. Moreover, the rapid decrease in *r*/*r*_C_ in the initial *T*/*T*_0_ < 0.4 illustrates that the free surface deformation develops rapidly with the creation of the entrapped air cavity, providing a context for interpreting the bubble size distribution. It should be noted that the present study employs two assumptions for the simplified mechanism of free-surface air entrainment: (1) the shape of entrapment deformation is hypothesized to be a Gaussian curve due to the relevant comparison results; (2) the air bubble entrainment occurs as a result of the entire process of local free surface entrapment deformation and evolving development. Other instability behaviours can also be observed to disturb and affect the entire air entrainment process, such as the adjacent deformed free surfaces, an air bubble transported upstream, and the interaction among other air–water structures. These microscopic behaviours are common for air–water flows^24^, which may have a specific instability influence acting in a particular period.

### Bubble distribution

One of the primary objectives of this study is to determine the entrained bubble scale within the interior of actively entrapped free surfaces. We achieve this objective by manually identifying and sizing bubbles in images similar to those shown in Fig. 1. To test the relationship between the entrapped deformation and entrained–air–bubble size, image sequences of the free-surface entrapment are examined. Although entrapment collapse events are frequently observed, image sequences are relatively rare. That is, 108 air–entrainment events are found in 36,000 video frames. This observation estimate is consistent with the idea that the entrapped free surface must reach a critical degree of deformation before fragmentation. The bubble size is characterized by the equivalent diameter *d*_ab_, which was obtained when the first frame with an intact and clear air–water interface appeared around a new bubble in the air-entrainment period. The size metrics are estimated for each event. Detailed data are available as the supplementary information.

**Figure 4:**
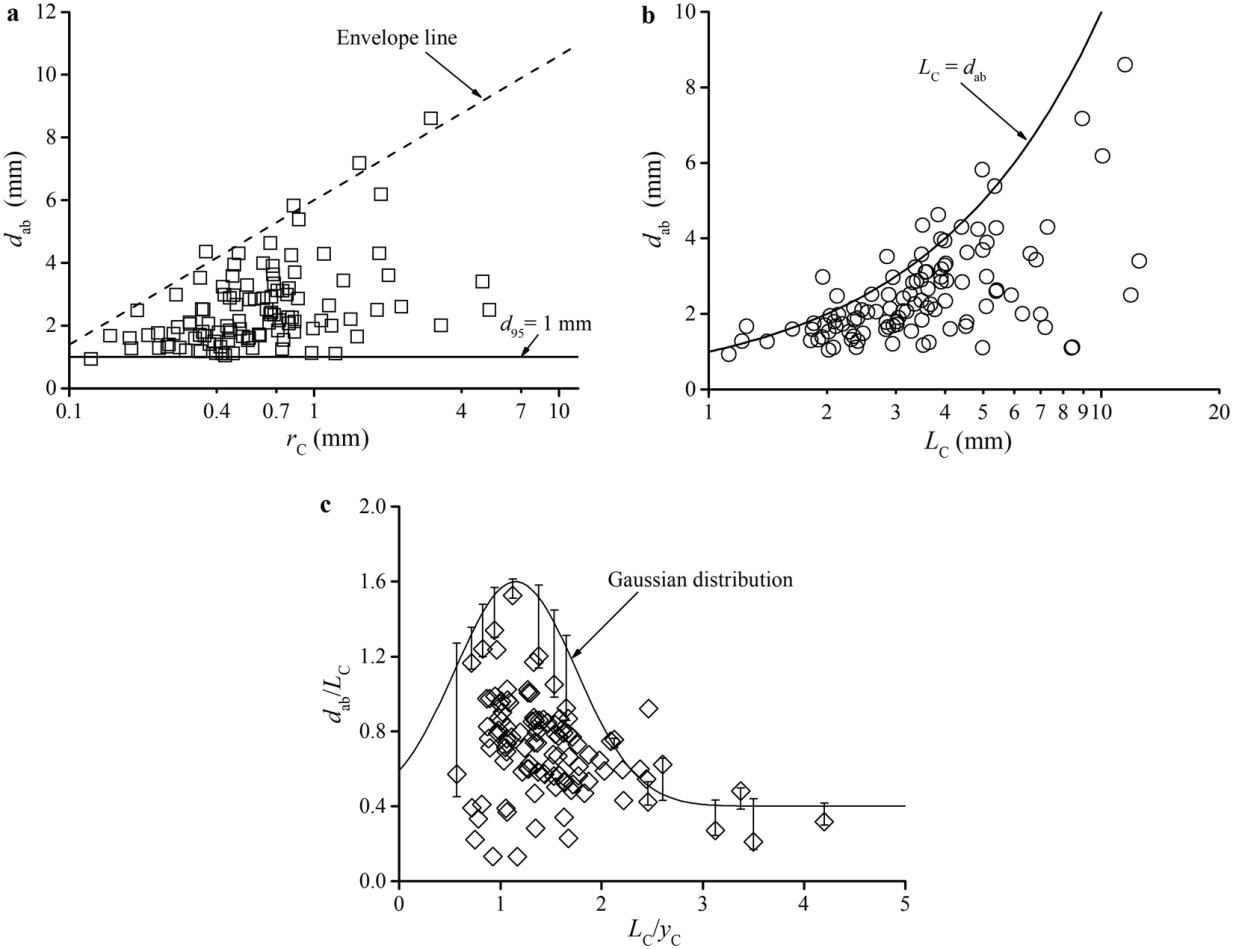
Bubble size spectrum entrained through the free surface. (**a**) The relationship between the critical radius of curvature *r*_C_ and the air bubble size *d*_ab_. The value of *d*_95_ is the critical bubble size on the Hinze scale. The envelope line of the maximum value of *d*_ab_ with ln(*r*_C_) follows a linear approximation *d*_ab_ = *m*·[ln(*r*_C_) + *n*·*d*_95_] with coefficients *m* = 2 and *n* = 3. (**b**) The relationship between the size scale of the entrapment deformation *L*_C_ and the air bubble size *d*_ab_. (**c**) Normal distribution of the bubble size as a function of *L*_C_/*y*_C_ (the solid line: *d*_ab_/*L*_C_ = 0.4 + 1.2·exp{−0.5·[(*L*_C_/*y*_C_ − 1.15)/0.6]^2^}).

**Figure 5:**
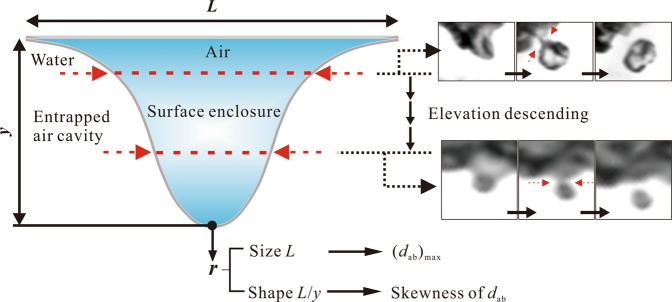
Illustration of the bubble entrainment affected by the surface enclosure.

Figure 4(a) shows scatter plots of the measured *d*_ab_ as a function of *r*_C_. From all the observed entrained bubbles, the probability of small-bubble creation is predominant, and the proportion of bubbles with size *d*_ab_ < 4 mm is approximately 97 events within 89.81% of the total observations. The critical radius of curvature *r*_C_ ranges from 0.1 to 10 mm, which characterizes two significant features: the critical radius skews with primarily small *r*_C_ < 1 mm and the increase in the maximum size of an air bubble for a certain entrapment condition with the increase in *r*_C_. Owing to the Gaussian–type assumption at the critical surface shape condition, two components characterize the deformation: (1) *L*_C_, which is the characterized size scale, and (2) *L*_C_/*y*_C_, which is the ratio of the shape width to the shape depth. The distribution of bubble sizes is determined by both the size scale and shape feature. In Fig. 4(b), compared to the size scale of the entrapment deformation, the bubble diameter is almost smaller, that is, *d*_ab_ < *L*_C_. The maximum size of an air bubble is approximately equal to *L*_C_. In Fig. 4(c), the dimensionless bubble size *d*_ab_/*L*_C_ exhibits a skewed distribution with respect to *L*_C_/*y*_C_, and most of the air bubbles form when *L*_C_/*y*_C_ is 1–2. Owing to the entrapment and enclosure mechanism of the free-surface air entrainment, the probability of the enclosure position at a lower elevation is higher than that at an upper elevation. This phenomenon is more prevalent for the “narrow–deep” shape, which is represented by a smaller *L*_C_/*y*_C_ ratio for identical *L*_C_ conditions than that for the “wide–shallow” shape, which is represented by a larger *L*_C_/*y*_C_ ratio (Fig. 5). Moreover, owing to the shearing force and complex movement of the adjacent free surface, a local entrapment deformation could scarcely develop to a large–sized *L*_C_ or a specific “narrow–deep” shape with small *L*_C_/*y*_C_. This prevalence determines the skewed distribution of the air bubble size.

## Discussion

For the free-surface air entrainment mechanism at the macroscopic level, the turbulent intensity, which is characterized by the fluctuation velocity normal to the free surface, should reach a critical threshold^25^. The present study shows that the free-surface entrapped deformation should be considered as the proximate cause of the air entrainment, resulting in newly created bubbles releasing into the water flow. This microscopic mechanism can be unified with the previous theory of the “bubble being created by a water droplet,” in which the falling droplets can be considered to be one of the dynamic sources causing the entrapment deformation of the local surface^26^. In addition, because of the random turbulence on the free-surface area, the entrapment and uplifting of the free surface should be treated equally without logical causation.

According to the observed behaviour of the surface entrapment generation and instability development, the physical mechanism can be extrapolated on the basis of a previous air–water interface mechanism. During the surface entrapment development process, the curvature at the boundary of the entrapped surface becomes sharply deformed in the late instable period. This can lead to a pressure difference between both sides of the air–water interface regions^24,27^. The pressure is higher in the water than in the air region, driving the interface from the water phase into the air phase. The pressure difference results in the appearance of the shrinkage. The probability of the shrinkage position at a lower elevation is higher than that at an upper elevation of the local cavity area. This prevalence determines the skewed distribution to a small air bubble size. However, due to the complex interaction among the turbulence, pressure difference and flowing adjacent free surface, the specific position where the shrinkage behaviour occurs is difficult to predict.

The central idea about free-surface air entrainment is that a bubble may be created if the turbulence across the entrapped free surface exceeds the restoring forces. The remnant of the turbulent kinetic energy Δ*E* transforms into the initial bubble kinetic energy and surface tension energy. When the bubble kinetic energy is zero, the largest bubble size with the greatest surface tension energy can be simply obtained as

2 $${({d}_{{\rm{ab}}})}_{{\rm{\max }}}=\sqrt{\frac{{\rm{\Delta }}E}{\pi \sigma }}$$

where *σ* is the fluid surface tension and Δ*E* is deduced from the flow conditions according to the method described in ref.^21^. On the other hand, based on the Hinze scale for the air bubble size in shearing water flows, the critical size *d*_95_, is predicted by the following procedure^28,29^

3 $${d}_{95}=0.725\cdot {[{(\frac{\sigma }{{\rho }_{w}})}^{3}\cdot {(\frac{1}{g{S}_{{\rm{f}}}V})}^{2}]}^{\frac{1}{5}}$$

where *S*_f_ is the energy slope, *V* is the mean flow velocity, *g* is the gravity acceleration constant, and *ρ*_w_ is the fluid density. *d*_95_ is the size for which 95% of the air is contained in bubbles of this size or smaller. This critical size represents the most stable bubble, which does not fragment in shearing flow.

The theoretical largest and smallest bubble sizes are (*d*_ab_)_max_ = 38.6 mm and *d*_95_ = 1 mm, respectively, as calculated from Eqs (2) and (3) based on the present flow conditions. The experimental observation showed the largest and smallest bubble sizes to be 8.6 and 0.9 mm, respectively. Clearly, most entrained bubbles from free-surface air entrainment tend to fracture into smaller bubbles under the turbulence and shearing effect. These research findings combined with methods of free surface dynamics and kinematic theories facilitate the prediction of the formation of bubble flow and the related bubble–size distribution.

According to the present experiments and analysis, the free-surface air entrainment can be characterized as the air–water interface instability. The main forces acting on the local surface perturbation in the turbulent open channel flow include the turbulent stresses, surface tension and gravity^30^. The surface tension and gravity forces are the constraining forces that can overcome the turbulent stress effect, which is significant for the sub–millimetre air–water interface deformation in two–phase multiphase flows^31^. In terms of the viscosity, a continuous interaction between the viscosity and other forces can be large at the air–water stratification layer^4^, which will constitute a more complex contribution for the local surface instability generation. The fluid mechanism is a continuous interaction among multiple forces at the air–water stratification layer. In further research, a significant and relevant point to address will be to fully characterize the air–water interface interaction and an accurate estimation of the specific forces. The instability criterion requires a formulation based on the detailed observation in the air–water interface behaviour, reaching a universal model for a good understanding of the air–water flow structures.

In conclusion, this study is an attempt to extend free-surface air entrainment into the broad topic of fluid mechanics of an open channel flow. The simplified process of surface deformation confirms that the instability behaviour of the local air–water interface results in the onset of bubble entrainment. The deformation intensity develops gradually, and the variation in the radius of curvature over time from the incipient entrapment generation to the instability collapse follows a power–law scaling. Due to the shrinkage of the surface instability behaviour, a single bubble forms as the shrinking surface becomes enclosed. The entrained bubble size depends on both the size scale and the deformation shape of the free surface in the instable period. The entrapped width of the deformed surface determines the largest bubble size, while the high probability of the appearance of shrinkage at the low entrapped position results in the skewed distribution to a small bubble size. However, detailed air–water interface interactions among multiple instability forces still have not been determined. Further research is necessary to elucidate the instability criterion at the dominant length scale of the surface deformation. A model dominated by the interface instability could lead a new understanding of multiphase flow generation.

## Methods

### Experiment

According to previous studies on the inception point of self–aeration in an open channel flow, the critical flow velocity is approximately 3–6 m/s, at which the weak air–water mixture can be observed in the model flows. Moreover, to obtain a clear air–water interface in the free-surface area, the flow velocity needs to be relatively low to reduce the interaction of the surface tension and the sidewall effect. The experiment is conducted in a chute with a width of 0.4 m and height of 0.5 m with a 36° slope. The water flow is supplied through the circulating supply system and is controlled by a flow inlet tank and a rectangular thin weir. In the test region, the mean water flow velocity is *V* = 5.5 m/s, and the depth is *H* = 4.4 cm. The flow Reynolds number *R*e = *VH*/*ν* and Weber number *W*e = *ρV*^2^*H*/*σ* are obtained to be 1.8 × 10^5^ and 1.8 × 10^4^, respectively, where *υ* is the kinematic viscosity, *ρ* is the water density, and *σ* is the surface tension. A high-speed camera system is used to measure the free-surface deformation from a two–dimensional view. The events are imaged 60 cm from the glass sidewall of the flume. To simplify the air–water interface images and facilitate the image processing, an LED plane light source is placed parallel to the imaging plane of the camera, supplying an even illumination intensity.

### Image analysis

The MotionPro Y3-class camera (Integrated Design Tools Inc., USA), with an image area of 1280 (width) × 344 (height) pixels, is focused on a plane 3 mm away and is positioned inside of the flume wall near the camera. A lens (Nikon AF Nikkor 24–85 mm) is controlled perpendicularly to the sidewall and is adjusted to an elevation identical to the free surface of the flowing water. Image sequences are captured at 1/3000 s with an image magnification factor of 0.25 mm/pixel and a shutter speed of 0.23 μs. To determine the length size, a ruler is placed at the focus plane to calibrate the conversion factor between pixels and millimetres. The images are first smoothed by a median filter, and the difference in the permeability between the distinct layers of air (non-illuminated pixels) and water (illuminated pixels) is considered to be a suitable characteristic for defining the boundaries between layers^32–34^. After the binary image is obtained from the grey image based on a suitable threshold, the entrapment surface parameters, including the width *L* and depth *y* scales and the bubble size of the 2D projected image, are determined according to the quantity of the non-illuminated columns of pixels^35–38^. In the flowing free-surface region, it is natural that the local surface entraps and deforms asymmetrically, affected by both the main flow velocity and local turbulent velocity. In a previous study^21^, asymmetric deformations bending both upstream and downstream were observed for different flow velocity conditions (4.2–7.6 m/s). For the image analysis, the entrapped cavity requires a rotation of the horizontal plane based on the inclined angle between the width dimension of the local entrapment and the streamwise direction. Because of the irregular spherical shape of a single air bubble, its size is characterized by an equivalent diameter *d*_ab_, where each bubble is considered to be a spherical particle with an identical area.

## Supplementary information

Dataset 1

## Data Availability

The raw data are available on request from the corresponding authors.
