# Supplementary material for: Bubble formation and scale dependence in free-surface air entrainment
Source: Sci Rep. 2019 Jul 29;9:11008. doi: 10.1038/s41598-019-46883-5 (PMC6662671; doi:10.1038/s41598-019-46883-5)
Supplement: Supplementary file 1 — Dataset 1 [file 41598_2019_46883_MOESM1_ESM.docx]

**Bubble formation and scale dependence in free-surface air entrainment**

Wangru Wei, Weilin Xu, Jun Deng*, Zhong Tian & Faxing Zhang

State Key Lab of Hydraulic and Mountain River Engineering, Sichuan University, Chengdu, Sichuan 610061, PR China.

Correspondence and requests for materials should be addressed to J.D. (Email address: djhao2002@scu.edu.cn)

**Supplementary material – size scale and shape characteristics for different radii of curvature**

**Supplementary Figure 1.**  Analysis process of free-surface entrapment deformation. Free-surface entrapment deformation during the air-bubble entrainment appears with a certain angle *θ* with the horizontal. In the image analysis process, a reconstruction entrapped cavity needs a (a) clockwise or (b) anticlockwise rotation before Gauss-type identification and size scale analysis.







**Supplementary Figure 2.** Typical shape characteristic of entrapment deformation. Radius of curvature at the apex of the entrapped surface *r*_C_ correlates well with both (a) size scale *L*_C_ and (b) shape character *L*_C_/*y*_C_, indicating the Gaussian-type assumption for free-surface entrapment.

**Supplementary Table 1.** Detailed parameters of total 108 air entrainment cases.

| **No.** | ***L*_C_ (mm)** | ***y*_C_ (mm)** | ***r*_C_ (mm)** | ***d*_ab_ (mm)** | **No.** | ***L*_C_ (mm)** | ***y*_C_ (mm)** | ***r*_C_ (mm)** | ***d*_ab_ (mm)** |
| --- | --- | --- | --- | --- | --- | --- | --- | --- | --- |
| 1 | 1.13 | 1.29 | 0.12 | 0.93 | 55 | 3.48 | 2.26 | 0.67 | 2.91 |
| 2 | 1.22 | 0.79 | 0.23 | 1.28 | 56 | 3.49 | 2.29 | 0.66 | 2.36 |
| 3 | 1.25 | 1.33 | 0.15 | 1.67 | 57 | 3.49 | 3.27 | 0.47 | 3.57 |
| 4 | 1.41 | 1.38 | 0.18 | 1.27 | 58 | 3.49 | 2.14 | 0.71 | 1.84 |
| 5 | 1.64 | 1.89 | 0.18 | 1.59 | 59 | 3.51 | 4.25 | 0.36 | 4.35 |
| 6 | 1.82 | 1.47 | 0.28 | 1.29 | 60 | 3.52 | 4.50 | 0.34 | 1.17 |
| 7 | 1.84 | 1.29 | 0.33 | 1.59 | 61 | 3.57 | 2.14 | 0.75 | 3.10 |
| 8 | 1.85 | 1.86 | 0.23 | 1.76 | 62 | 3.59 | 2.26 | 0.71 | 3.11 |
| 9 | 1.90 | 1.79 | 0.25 | 1.32 | 63 | 3.60 | 1.51 | 1.07 | 2.16 |
| 10 | 1.95 | 1.85 | 0.26 | 1.39 | 64 | 3.62 | 3.40 | 0.48 | 2.66 |
| 11 | 1.95 | 1.74 | 0.27 | 2.98 | 65 | 3.64 | 2.24 | 0.74 | 1.24 |
| 12 | 1.98 | 1.43 | 0.34 | 1.70 | 66 | 3.67 | 2.07 | 0.82 | 2.25 |
| 13 | 2.02 | 1.18 | 0.43 | 1.04 | 67 | 3.77 | 2.13 | 0.83 | 2.12 |
| 14 | 2.02 | 0.95 | 0.54 | 1.53 | 68 | 3.85 | 2.79 | 0.66 | 4.63 |
| 15 | 2.04 | 1.87 | 0.28 | 1.95 | 69 | 3.90 | 3.07 | 0.62 | 3.99 |
| 16 | 2.08 | 1.28 | 0.42 | 1.11 | 70 | 3.91 | 4.43 | 0.43 | 2.98 |
| 17 | 2.09 | 1.55 | 0.35 | 1.79 | 71 | 3.91 | 2.22 | 0.86 | 2.86 |
| 18 | 2.11 | 1.55 | 0.36 | 1.67 | 72 | 3.92 | 2.43 | 0.79 | 3.19 |
| 19 | 2.12 | 2.97 | 0.19 | 2.48 | 73 | 3.99 | 4.21 | 0.47 | 3.94 |
| 20 | 2.14 | 1.30 | 0.44 | 1.98 | 74 | 4.00 | 3.75 | 0.53 | 3.28 |
| 21 | 2.19 | 2.25 | 0.27 | 1.73 | 75 | 4.01 | 2.92 | 0.69 | 2.34 |
| 22 | 2.28 | 1.46 | 0.45 | 1.53 | 76 | 4.02 | 2.94 | 0.69 | 3.33 |
| 23 | 2.31 | 1.61 | 0.42 | 1.32 | 77 | 4.04 | 4.49 | 0.45 | 2.88 |
| 24 | 2.34 | 1.83 | 0.37 | 1.41 | 78 | 4.12 | 3.91 | 0.54 | 1.60 |
| 25 | 2.34 | 0.95 | 0.72 | 2.16 | 79 | 4.41 | 4.93 | 0.49 | 4.30 |
| 26 | 2.35 | 1.52 | 0.45 | 1.84 | 80 | 4.43 | 4.28 | 0.57 | 2.84 |
| 27 | 2.38 | 1.77 | 0.40 | 1.12 | 81 | 4.52 | 4.24 | 0.60 | 1.67 |
| 28 | 2.40 | 1.28 | 0.56 | 1.28 | 82 | 4.55 | 6.33 | 0.41 | 1.78 |
| 29 | 2.46 | 2.43 | 0.31 | 2.10 | 83 | 4.56 | 3.81 | 0.68 | 3.62 |
| 30 | 2.47 | 1.95 | 0.39 | 1.48 | 84 | 4.86 | 3.65 | 0.81 | 4.24 |
| 31 | 2.54 | 2.59 | 0.31 | 2.05 | 85 | 4.98 | 3.75 | 0.83 | 5.82 |
| 32 | 2.60 | 2.43 | 0.35 | 2.52 | 86 | 4.99 | 6.66 | 0.47 | 1.11 |
| 33 | 2.67 | 2.41 | 0.37 | 2.06 | 87 | 4.99 | 3.73 | 0.83 | 3.69 |
| 34 | 2.83 | 1.85 | 0.54 | 1.59 | 88 | 5.10 | 2.30 | 1.41 | 2.20 |
| 35 | 2.84 | 2.20 | 0.46 | 1.78 | 89 | 5.11 | 4.20 | 0.78 | 2.99 |
| 36 | 2.85 | 2.95 | 0.34 | 3.52 | 90 | 5.12 | 4.82 | 0.68 | 3.89 |
| 37 | 2.87 | 2.01 | 0.51 | 1.65 | 91 | 5.36 | 4.13 | 0.87 | 5.38 |
| 38 | 2.88 | 2.92 | 0.35 | 2.51 | 92 | 5.40 | 1.60 | 2.28 | 2.60 |
| 39 | 2.90 | 2.13 | 0.49 | 2.14 | 93 | 5.41 | 3.33 | 1.10 | 4.28 |
| 40 | 2.94 | 3.61 | 0.30 | 1.21 | 94 | 5.42 | 3.19 | 1.15 | 2.63 |
| 41 | 2.95 | 2.31 | 0.47 | 2.97 | 95 | 5.90 | 2.40 | 1.81 | 2.50 |
| 42 | 2.95 | 5.19 | 0.21 | 1.69 | 96 | 6.30 | 1.50 | 3.31 | 2.00 |
| 43 | 3.02 | 1.37 | 0.83 | 1.80 | 97 | 6.60 | 2.70 | 2.02 | 3.60 |
| 44 | 3.02 | 1.91 | 0.60 | 1.71 | 98 | 6.82 | 4.41 | 1.32 | 3.43 |
| 45 | 3.05 | 1.17 | 0.99 | 1.90 | 99 | 7.01 | 5.20 | 1.18 | 1.99 |
| 46 | 3.13 | 1.87 | 0.66 | 2.08 | 100 | 7.20 | 4.30 | 1.50 | 1.65 |
| 47 | 3.13 | 1.83 | 0.67 | 2.41 | 101 | 7.30 | 3.60 | 1.85 | 4.30 |
| 48 | 3.20 | 1.61 | 0.79 | 2.06 | 102 | 8.40 | 7.20 | 1.23 | 1.10 |
| 49 | 3.26 | 2.07 | 0.64 | 2.52 | 103 | 8.45 | 9.12 | 0.98 | 1.12 |
| 50 | 3.28 | 1.79 | 0.75 | 1.54 | 104 | 8.95 | 6.53 | 1.53 | 7.17 |
| 51 | 3.31 | 2.52 | 0.54 | 2.83 | 105 | 10.09 | 6.77 | 1.88 | 6.18 |
| 52 | 3.35 | 1.79 | 0.78 | 2.26 | 106 | 11.50 | 5.50 | 3.01 | 8.60 |
| 53 | 3.36 | 3.33 | 0.42 | 3.23 | 107 | 11.90 | 3.40 | 5.21 | 2.50 |
| 54 | 3.38 | 2.31 | 0.62 | 2.87 | 108 | 12.50 | 4.00 | 4.88 | 3.40 |
